# Mechanisms and Applications of Bacterial Inoculants in Plant Drought Stress Tolerance

**DOI:** 10.3390/microorganisms11020502

**Published:** 2023-02-17

**Authors:** Priscila Pires Bittencourt, Alice Ferreira Alves, Mariana Barduco Ferreira, Luiz Eduardo Souza da Silva Irineu, Vitor Batista Pinto, Fabio Lopes Olivares

**Affiliations:** 1 Laboratório de Biologia Celular e Tecidual, Centro de Biociências e Biotecnologia, Núcleo de Desenvolvimento de Insumos Biológicos para a Agricultura (NUDIBA), Universidade Estadual do Norte Fluminense Darcy Ribeiro (UENF), Campos dos Goytacazes 28013-602, RJ, Brazil; 2 Laboratório de Biotecnologia, Centro de Biociências e Biotecnologia & Unidade de Biologia Integrativa, Setor de Genômica e Proteômica, Universidade Estadual do Norte Fluminense Darcy Ribeiro (UENF), Campos dos Goytacazes 28013-602, RJ, Brazil

**Keywords:** bioinoculant, sustainable agriculture, PGPB, abiotic stress, endophytic bacteria, water-use efficiency

## Abstract

Agricultural systems are highly affected by climatic factors such as temperature, rain, humidity, wind, and solar radiation, so the climate and its changes are major risk factors for agricultural activities. A small portion of the agricultural areas of Brazil is irrigated, while the vast majority directly depends on the natural variations of the rains. The increase in temperatures due to climate change will lead to increased water consumption by farmers and a reduction in water availability, putting production capacity at risk. Drought is a limiting environmental factor for plant growth and one of the natural phenomena that most affects agricultural productivity. The response of plants to water stress is complex and involves coordination between gene expression and its integration with hormones. Studies suggest that bacteria have mechanisms to mitigate the effects of water stress and promote more significant growth in these plant species. The underlined mechanism involves root-to-shoot phenotypic changes in growth rate, architecture, hydraulic conductivity, water conservation, plant cell protection, and damage restoration through integrating phytohormones modulation, stress-induced enzymatic apparatus, and metabolites. Thus, this review aims to demonstrate how plant growth-promoting bacteria could mitigate negative responses in plants exposed to water stress and provide examples of technological conversion applied to agroecosystems.

## 1. Introduction

Climate changes and the increase in global demand for food, fiber, and energy are two pressure factors that directly impact agricultural systems, compromising food security and agroecosystem sustainability [1]. In addition to a century of fossil fuel demands of industrialization associated with urban activities, deforestation, and intensive land use have increased the emission of greenhouse gases, thus identifying food chain production activities as having a significant responsibility for climate change trends [2].

Accordingly, temperature and rainfall regime changes are one of the main constraints that will increasingly affect food production in this century. Furthermore, about 50% of the loss in agricultural productivity is related to abiotic factors, while biotic factors account for about 30% [1,2]. Thus, one of the main challenges for the future of agriculture is to increase yield or mitigate crop losses using techniques and strategies under global climate change scenarios and economic constraints [3].

Any adverse change in plant physiology due to an external factor that modifies its balance can be defined as stress. Several abiotic stressors, such as temperature, drought, salinity, flooding, and heavy metals, affect plant morphology, physiology, biochemistry, and gene regulation leading to reductions in crop productivity. Plant breeding has been widely used to select genotypes tolerant to diverse abiotic stresses, with remarkable efforts to develop drought-tolerance varieties under an integrative perspective that combines conventional and modern breeding tools [4]. In parallel, several studies have been conducted to unravel the molecular bases and the morpho-physiological traits related to improving drought tolerance [5,6,7,8,9,10,11].

Plant growth-promoting bacteria (PGPB) have been proposed to mitigate environmental stresses in two primary acting modes including plant water conservation mechanisms and protection–recovering mechanisms. Consequently, there is an increased interest in converting the scientific knowledge related to drought-mitigating bacteria into a sustainable solution for agroecosystems. Currently, microbial inoculants represent the most feasible biological technology to fulfill plant growth requirements in association with crop protection against biotic and abiotic constraints [12,13]

This review will cover the core mechanisms displayed to alleviate plant water stress mediated by bacterial inoculants that involve complex molecular machinery mediated by phytohormonal signalizing, an induced enzymatic pool, and metabolites to increase soil water accessibility and reduce plant water loss. Moreover, it will highlight the coordinated combination of catalytic proteins and metabolites employed to prevent plant cell damage and trigger repair systems that enhance water scarcity tolerance. In addition, past and present strategies, technological applications, and prospects for bacterial inoculants to mitigate drought stresses will be considered.

## 2. Core Mechanisms of Drought Tolerance of Plants

Drought is considered one of the greatest threats to global agricultural quality and productivity, limiting plant species’ growth and development, and is defined as a meteorological term characterized by sub-normal rainfall over a long period that compromises the necessary soil moisture for a given crop at a given time. In this context, a decrease in water availability has a deleterious effect on growth and development, influencing the life cycle of plants [14].

During periods of drought, there is a decrease in the water potential of the soil and, consequently, a decrease in the water potential of the plant. Thus, the response of plants to drought is a determining factor in maintaining balance along the soil–plant–atmosphere continuum and a complex phenomenon marked by a series of molecular, biochemical, and physiological changes. Stomatal closure is the first response mechanism to prevent leaf cavitation and embolism [15,16]. Hochberg et al. [17] conducted studies with grape leaves and demonstrated that the stomata closed completely before observing cavitation. Consequently, there is a simultaneous decrease in CO_2_ influx, directly influencing photosynthetic capacity, while photorespiration increases. Reducing carbon incorporation into plant biomass under water scarcity affects plant growth and energy needs to drive plant drought responses related to cell protection and damage restoration.

An interplay between two main approaches is required to increase plant resilience to water stress. One involves a combination of morphophysiological mechanisms to increase the plant water status that is mainly orchestrated by a hormonal signaling network (i.e., auxin, cytokinin, gibberellin) [18,19,20]. In addition to being essential for growth and development, they play an important role in signaling stress. For example, abscisic acid (ABA), the stress hormone, is significantly detected during drought events. It is responsible for promoting stomatal closure and regulating several genes responsible for dehydration tolerance [21,22].

Another strategy to retain water in the plant body is to cope with plant cell osmolarity modulation by the intracellular solute concentration (soluble sugars, sorbitol, proline, and glycine) that increases to maintain cell turgor, a process called an osmotic adjustment [23]. In addition to maintaining turgor pressure, these solutes protect plant cells from the effects of toxic by-products formed during drought [23]. Initial evidence of osmotic adjustment has been reported in pea roots [24] and sorghum [25]. Later, several studies demonstrated the maintenance of a plant’s turgor due to the osmotic adjustment mechanism [26,27,28,29,30]. However, it is essential to note that the degree of response in the osmotic adjustment depends on the plant species/cultivar and the duration of the stress event [27].

A technological derivation of the accumulation in osmolytes results from the application of organic compounds as the foliar spray increases the tolerance of plants under stress conditions, for example, the application of L-ornithine in sugar beet (*Beta vulgaris* var. *saccharifera* L.) [31], *Catharanthus roseus* [32], *Brassica* spp. [33], and *Raphanus sativus* L. [34].

Water-conservation approaches are associated with protective and repairing machinery [10]. Similar to other abiotic stresses, water deficit results in an excessive reduction in the electron transport chain (ETC), which increases plant tissue photo-oxidation [35]. As a result, the enzymatic Rubisco (EC 4.1.1.39-ribulose-bisphosphate carboxylase) activity declines, and the photosystem II (PSII) membrane complex is damaged, resulting in the repression of photosynthetic activity [36]. Consequently, a significant photoprotective response leads to the dissipation of excess energy as heat, known as the non-photochemical quenching (NPQ) of chlorophyll fluorescence [37].

Plants also display a series of molecular and biochemical mechanisms in response to drought. One of these well-studied mechanisms is the induction of the production of reactive oxygen species (ROS) that can result in membrane peroxidation and lead to oxidative damage, impairing cellular functions. Therefore, plants developed an antioxidant defense system based on various enzymes (superoxide dismutase (SOD), peroxidase (POX), catalase (CAT), and glutathione reductase (GR)) that alleviate oxidative damage [38].

There is, therefore, a positive correlation between drought tolerance and the antioxidant response. More tolerant plant species present better antioxidant responses, consequently increasing the activity of antioxidative enzymes, protecting the plant from oxidative damage. Meanwhile, species more sensitive to drought do not show changes in such enzyme activity machinery [39,40,41].

Several studies have demonstrated the increased activity of antioxidant enzymes in response to drought. These antioxidant responses can vary between different cultivars, as observed in rice *Oryza sativa* L. [39], *Triticum aestivum* L. [42], and *Hordeum vulgare* L. [43]. These authors demonstrated that cultivars more tolerant to drought have lower oxidative stress and ROS production levels than cultivars that are more sensitive to drought. Therefore, these differences observed between more sensitive and drought-tolerant genotypes help to understand new stress response mechanisms and to produce more resistant crops using breeding and genetic engineering approaches [43]. It is worth noting that ROS are essential for maintaining cellular processes but an above-normal amount has a toxic effect. Therefore, it is necessary to maintain homeostasis of ROS levels, which is above the cytostatic level but below the cytotoxic level [35,36].

In addition to the molecular, biochemical, and physiological responses triggered by drought in plants, the interaction with microbial communities found in the rhizosphere and root–shoot surface (epiphytes) and inner tissues (endophytes) can help to enhance plant fitness under environmental stressors [44,45]. In natural conditions, plants and bacteria are closely related; studying these interactions helps to understand and boost the underlying mechanisms of tolerance to drought.

## 3. Plant Growth-Promoting Bacteria

Soil bacteria communities represent the most diverse, abundant, and physiologically active group of organisms, with bacterial phylotypes ranging from 10^2^ to 10^6^ per gram of soil [46]. This vast diversity represents the soil microbiome that plays a pivotal role in the biogeochemical process and nutrient cycling, serving as a “seed bank” of species richness [47,48]. When seeds or other reproductive plant structures are sowed, root development through the soil creates a new ecological niche called the rhizosphere. It is described as a soil perimeter around the root axis enriched with various carbon exudates (e.g., organic acids, amino acids, sugars, flavonoids), the mucilaginous matrix, and detached root cap cells that create a high-activity microbial environment with plant recruitment of more adapted rhizosphere colonization competent taxa [49,50]. 

Potentially beneficial or pathogenic microorganisms occupy the rhizosphere and compete in the colonization of plant tissues, modulating nutrient flux throughout the soil–plant system, thus affecting the growth and development of plants [51]. Among such microorganisms, bacteria have been pivotal as the most frequent and active live fraction interacting with the plant host [52]. Among the benefits to the host, several reports show biofertilizer, biostimulant, and bioprotection effects characterized as plant growth promotion, protecting against pathogens, and mitigating various environmental stresses [13,53,54,55]. Such a broad group, known as plant growth-promoting bacteria (PGPB), encompasses several genera such as *Pseudomonas*, *Enterobacter*, *Bacillus*, *Variovax*, *Klebsiella*, *Paraburkholderia*, *Azospirillum*, *Herbaspirillum*, *Gluconacetobacter*, *Serratia*, *Azotobacter*, among others [56,57].

**Figure 1:**
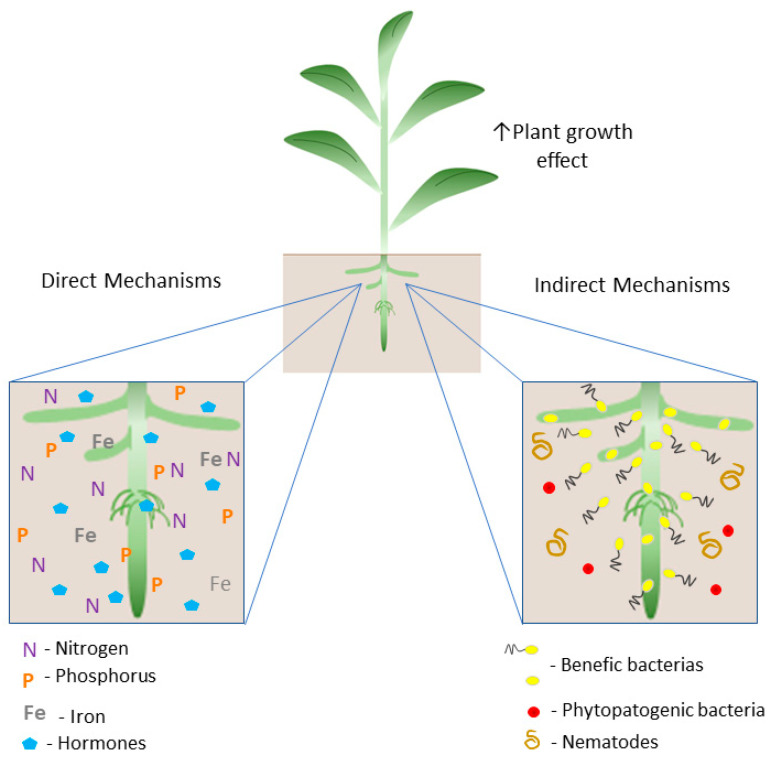
Schematic illustration of the plant growth promotion mechanisms by PGPB. The direct mechanism includes biological nitrogen fixation (BNF) by the activity of the nitrogenase enzyme complex; solubilization of inorganic phosphate in the soil; production of siderophores, increasing the availability of iron, and the production of hormones such as auxins, gibberellins, and cytokinin that modulate the hormonal balance of the plant host. Indirect mechanisms are related to the occupation of niches by PGPB and the production of substances with repelling functions, preventing colonization by phytopathogens and nematodes.

These microorganisms, when associated with plants, promote plant growth through the direct mechanisms (1): biological nitrogen fixation; hormone production (indole-3-acetic acid (IAA), gibberellic acid (GA3), and cytokinin, such as zeatin (Z)); and the acquisition of essential nutrients (phosphorus and iron); and (2) indirect mechanisms: related to biocontrol, through the mitigation of damage caused by pathogens and/or environmental stresses [13,44,51,53,54,55,56,57] (Figure 1).

It is known that there is a positive response from this interaction both to promote growth and alleviate stress in plants. In addition, several mechanisms have already been described in the responses and interactions between PGPB and plants to reduce the damage caused by environmental stress on plants. For example, the production of hormones (auxin, cytokinin, abscisic acid); the synthesis of exopolysaccharides and beneficial enzymes, such as 1-aminocyclopropane-1-carboxylate deaminase (ACC deaminase); the synthesis of trehalose and volatile organic compounds; and the responses related to osmoregulation. Highlights of the general mechanisms of drought resistance by PGPB are presented in Table 1.

## 4. Stress Tolerance Mechanisms Mediated by Plant Growth-Promoting Bacteria (PGPB)

### 4.1. Bacterial Phytohormones and Modulation of Plant Morpho-Physiological Traits

**Figure 2:**
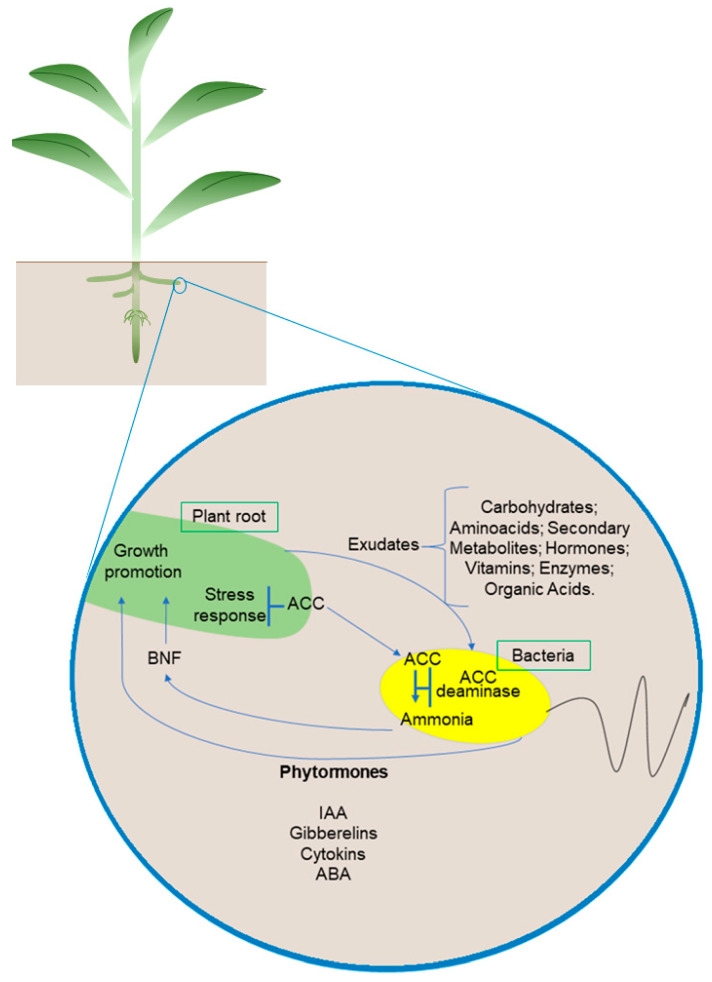
Microorganism–plant interaction. Plant roots and bacterial cells synthesize metabolites as substrates and signaling molecules. Microorganisms used as biofertilizers promote plant growth through biological nitrogen fixation, nutrient solubilization (phosphate and iron), and the production of hormones and other compounds. A dashed line indicates a positive relationship between plants and bacteria. Abbreviations: ACC, 1-aminocyclopropane-1-carboxylate; ACC deaminase, 1-aminocyclopropane-1 carboxylate deaminase; BNF, biological nitrogen fixation. Organisms and cells are not to scale.

The promotion of plant growth through beneficial microorganisms is mainly related to a complex network of plant hormones. Plant hormones are essential for growth, development, and responses to biotic and abiotic stimuli [68]. The interaction between PGPB and plants is associated with a series of changes, especially with hormonal homeostasis. Some microorganisms can produce and/or modulate several hormonal classes associated with changes in the concentration, location, and signaling of hormones, consequently affecting their balance in the plant [68,69,70]. Thus, the promotion of plant growth through beneficial microorganisms is mainly related to a complex network of plant hormones (Figure 2).

Auxin is one of the best-studied plant hormones and has several reported functions related to plant cell division, expansion, and differentiation [71]. For example, auxinic activity stimulates the germination of seeds and tubers; increases the rate of xylem and root development; controls vegetative growth processes; initiates lateral and adventitious root formation; mediates responses to light, gravity, and flowering; and affects photosynthesis, pigment formation, the biosynthesis of various metabolites, and the resistance to stressful conditions [72]. In addition, several PGPB regulate the auxin balance and thus change the root growth rates and architecture [69,72,73,74]. 

Changes in root anatomy and biochemistry mediated by phytohormonal modulation are one of the well-recognizable microbial inoculant’s actions on crop agroecosystems [51,53,55,69,73]. Interestingly, such plant-growth promotion effects are pivotal in increasing water availability under environmental scarcity when the soil matrix water potential is low. In such circumstances, the water uptake is enhanced by a combination of absorptive root structures, such as increasing lateral root formation (root ramification), root hair density, and length (specific surface enhancement) with a consequent increase in root surface, volume, and biomass. Furthermore, water influx enhancement involves changes in the organization pattern of epidermal, cortical, and vascular root tissue systems that modulate the root system’s hydraulic conductivity [75]. Among them are the number and arrangement of cell layers, apoplastic resistivity to water flux, metaxylem number, diameter and distribution, and increased density of water channel transmembrane transporters (i.e., aquaporin). In addition, the auxin signaling pathway activates electrogenic transmembrane pumps (P-type H^+^-ATPase at the plasma membrane and V-type H^+^-ATPase at the vacuolar membrane) that generate an electrochemical gradient to accommodate the secondary transport of nutrients [76]. Furthermore, the P-type pump activity acidifies the apoplast microenvironment of the recent-divided cells near the root meristematic tip, which is responsible for cell and tissue expansion and primary growth of the root axis [77]. In summary, bacteria inoculation modulates auxin signaling and balance, promoting plant growth under appropriate water availability or alleviating the deleterious effect of water scarcity on plant growth or development.

Abscisic acid (ABA), a stress hormone, is significantly detected during drought events. ABA promotes stomatal closure and regulates several genes responsible for tolerance to dehydration [22]. Cohen and collaborators [78] studied bacteria of a specific genus and suggested that the bacterium can provide the plant with exogenous ABA, which could explain the plant’s improved ability to deal with some abiotic stresses. Under field conditions in Brazil, maize crop plants inoculated with *Azospirillum brasilense* strains Ab-V5 and Ab-V6 showed better growth recovery after rainfall from a prolonged drought than non-inoculated plants [79]. 

In addition, other studies have shown that during the microorganism–plant interaction, there is a control on the hormonal balance of ABA in plants, thus promoting plant growth even under stressful conditions. For example, Curá and collaborators [67] demonstrated that inoculation with the bacteria *Azospirillum* and *Herbaspirillum* in maize plants directly affects molecular, biochemical, and physiological processes. Furthermore, Salomon and collaborators [80] reported that PGPB inoculation induces the accumulation of ABA in plants of *Vitis vinífera*.

The complex crosstalk involving the modulation of auxin and ABA in bacterial-inoculated plants under water scarcity generates a convergent action mechanism for water use efficiency. On one side, auxin signaling pathways increase phenotypic traits related to the uptake and transport of water. On the other side, the ABA signal cascade operates to reduce water losses through the transpiration process. Such dual-mode action increases plant tissue water content under stressful conditions. Under severe drought, plants show a survival phenotype, and under mild drought, microbial inoculation promotes growth and development compared to non-inoculated stressed plants.

It is worth mentioning that some experimental assays under greenhouse and field conditions have shown significant fresh biomass increase despite the non-significant dry biomass accumulation related to plant response to microbial inoculation. This suggested that bacteria inoculation increases the water content in the plant body and would benefit plant fitness in water-scarce environments [81].

The integrative role of the auxin–ABA signaling network that promotes water conservation in the plant cell and tissue cannot be considered apart from the osmoregulation mechanism. Nevertheless, considering the concept of plant phenotypic plasticity under harsh environmental conditions [82], changes in plant microstructure would increase the plant body’s ability to store and circulate water. Among them are the increased plant cell vacuolization, increased water storage specialized cells, and changes in the volume ratio of the apoplastic and symplastic compartments [75,83]. Furthermore, these integrative mechanisms of plant water conservation favor the induction of plant response enzymatic–metabolic machinery related to cell protection and damage restoration (i.e., ROS production associated with biological membranes and biomolecule damage).

Low soil water availability and a high atmospheric temperature impose a decrease in leaf water potential with a concomitant decrease in the stomatic conductance and transpiration rates and progressively lower photosynthetic rates until complete closure of the stomata, preventing water loss in the plant tissue coupled with no photosynthetic activity. There are some reports in the literature that, to some degree, bacteria inoculation can increase net photosynthetic activity compared to non-inoculated plants with similar stomatic conductance values [84,85,86,87,88]. Supposedly, the inoculated bacteria increase water use efficiency by enhancing carbon dioxide influx or reducing respiration rates with the same rate of water vapor lost from sub-stomatic chambers in leaf blades, also leading to an extra C-acquisition to fulfill the energetic requirements to restore cell homeostasis [89]. However, the underlying mechanism is still an open scientific question. 

Ethylene gas is another phytohormone with a pivotal role that can affect plant growth and development in several ways, including promoting root initiation, inhibiting root elongation, promoting fruit ripening, inducing flower wilt, stimulating seed germination, promoting leaf abscission, activating the synthesis of other plant hormones, and responding to biotic and abiotic stresses.

Under stressful conditions, a plant can increase ethylene synthesis as one of the response mechanisms. The immediate precursor of ethylene is 1-aminocyclopropane-1-carboxylate (ACC). The synthesis of this hormone begins with methionine, which will be converted to S-adenosylmethionine (SAM) via S-adenosylmethionine synthase (SAM synthase), while ACC synthase converts SAM to ACC. Thus, there is an increase in the concentrations of ACC and, consequently, ethylene levels. However, in high concentrations, this hormone inhibits the growth and yield of crops.

Some PGPB have the enzyme 1-aminocyclopropane-1-carboxylate deaminase (ACC deaminase). This enzyme was first characterized by Honma and Shimomura [90] and is directly involved in promoting plant growth under stressful conditions. The model proposed by Glick et al. [91] demonstrates that PGPB synthesize and secrete auxin, which is transported to seeds and roots, promoting plant growth in response to tryptophan. In addition, auxin can stimulate the activity of 1-aminocyclopropane-1-carboxylate synthase (ACC synthase) to convert S-adenosylmethionine into ACC. Part of this ACC can be exuded by the roots, returning to the bacteria, and hydrolyzed via ACC deaminase in ammonia and α-ketobutyrate.

**Figure 3:**
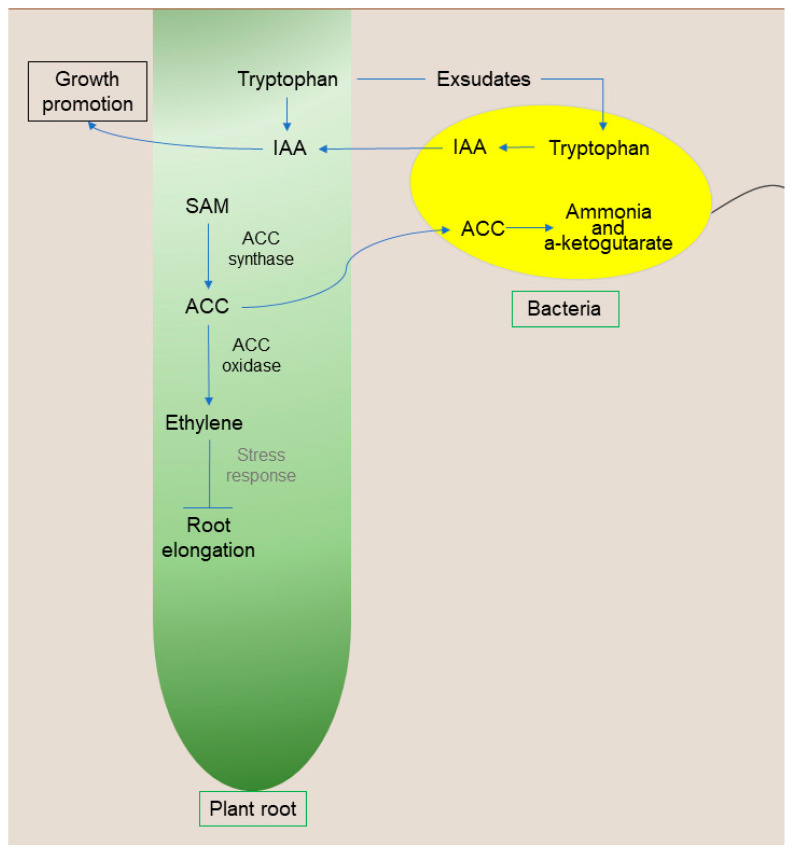
Schematic representation of bacteria with ACC deaminase activity. Abbreviations: AIA, auxin; SAM, S-adenosylmethionine; ACC, 1-aminocyclopropane-1-carboxylate; ACC deaminase, 1-aminocyclopropane-1-carboxylate deaminase; BNF, biological nitrogen fixation. Organisms and cells are not to scale.

Consequently, there is a decrease in the concentration of ACC outside the plant. To maintain the balance between ACC’s internal and external concentrations, the plant exudes more ACC. Since ACC is the immediate precursor of the hormone ethylene in plants, a reduction in this compound is directly related to a reduction in the level of ethylene [92], thus promoting plant growth even in limiting conditions. Furthermore, bacteria that present this enzyme enable the plant to become more resistant to biotic and abiotic stresses [92] (Figure 3). ACC deaminase activity is vital to promoting growth, especially in stress conditions. In this context, several studies are dedicated to demonstrating that the inoculation of bacteria capable of synthesizing ACC deaminase is an excellent growth promoter in plants under abiotic stress [60,64,91,92].

### 4.2. Osmoregulation: Bacterial Synthesis and Induced Accumulation in the Plant Host Cell

Plant growth-promoting bacteria can synthesize osmolytes that are secreted together with other exuded compounds. These osmolytes act synergistically with osmolytes synthesized by plants (such as glycine-betaine, soluble sugars, trehalose, and proline), act as osmoprotectants, and prevent cell damage caused by drought [93]. Thus, the accumulation of these osmolytes in plants, triggered by these microbes, influences an increase in tolerance to water stress [59,61,62,66,67]. 

Plants inoculated with PGPB may show an increase in the concentration of proline in water deficit conditions, thus conferring tolerance to stress and maintaining cell turgor and membrane stability and preventing the leakage of electrolytes. Thus, the increase in proline prevents oxidative damage in plants [28]. For example, the inoculation of *Arthrobacter* sp. and *Bacillus* spp. in pepper plants increases proline synthesis and accumulation [94]. Drought-tolerant strains of *Bacillus* spp. reduced the activity of antioxidant enzymes increased the plant’s biomass, and increased the relative content of water, proline, sugars, and free amino acids in sunflower (*Helianthus annuus* L.) and maize (*Zea mays* L.) [95]. *Arabidopsis thaliana* inoculated with *Azospirillum baldaniorum* strain Sp 245 (formerly named as *Azospirillum brasilense*) under water deficit conditions showed increased proline levels and relative water content, consequently improving the plants’ performance in drought conditions [78]. 

The increase in osmolyte contents in the cytoplasmatic compartment triggered by bacterial inoculants (secreted or plant-induced by microbes) reduces the osmotic pressure inside the plant cell, avoiding water efflux. Therefore, it works in an orchestrated connection mechanism displayed by the phytohormonal imbalance to modulate water balance and flux inside the plant body. Accordingly, the improved hydrated microenvironment allows photosynthetic activity recovery to maintain the molecular arsenal that combats subcellular compartment damages.

### 4.3. Bacterial Exopolysaccharides’ Self-Protection and Water-Retaining Properties

Exopolysaccharides (EPSs) are highly heterogeneous, high molecular weight polymers. They have many distinct monosaccharides, which are soluble in water and composed of sugar residues, and are secreted by microorganisms in the surrounding environment, found mainly in microbial cells in extreme environments [96]. Therefore, EPS synthesis is one of the most common self-protective mechanisms described for bacteria. The synthesis of this compound is a strategy used to grow, adhere to solid surfaces, and survive adverse conditions, representing 40% to 95% of the bacterial weight. In addition, they are essential for forming and maintaining the biofilm architecture, retaining water and absorbing nutrients, and increasing survival in harsh environments [97]. 

The advantages promoted by EPS synthesis favor both bacteria and plants under stressful conditions [96]. Thus, bacteria capable of synthesizing exopolysaccharides are fundamental for promoting plant growth in stressful conditions, e.g., drought, since this mechanism increases the soil’s water retention capacity. It is worth highlighting that these bacteria are more advantageous and have gained prominence in being used as bio-inoculants for plant tolerance to drought [98]. 

Studies have shown that bacteria of the genera *Bacillus*, *Pseudomonas*, and *Azospirillum*, among other microorganisms, are capable of secreting EPSs under water stress conditions and can confer tolerance to abiotic stress on plants [96,98]. In addition to changes in the root structure of plants, these compounds can act as an emulsifier and thus mitigate effects triggered by ROS. Furthermore, plants inoculated with bacteria capable of synthesizing EPSs have a more significant accumulation of proline, sugars, and free amino acids and increased plant biomass, leaf area, and protein content [99]. 

### 4.4. Bacterial Volatile Organic Compounds as Signals for Drought Bioprotection

Several mechanisms have already been described and highlight the potential of these microorganisms to increase crop yields. In addition, PGPB mechanisms can also produce gaseous organic molecules called volatile organic compounds (VOCs) [98].

VOCs are low molecular weight lipophilic compounds (<300 mol. L^−1^) emitted by plants during development, as well as in response to biotic and abiotic stresses [100]. For example, plants in stressful conditions emit VOCs through the leaves, such as isoprenoids, and improve plant resistance since the emissions of these compounds provide mitigation of the effects caused by ROS and increase the protection of cell membranes [101]. Microorganisms can also emit VOCs and act as signal molecules in the rhizosphere over short and long distances. This mechanism was first reported to promote the growth of *Arabidopsis thaliana* inoculated with *Bacillus subtilis* [102]. 

Several genera of bacteria can synthesize these compounds, such as *Burkholderia*, *Pantoea*, *Serratia* and *Chromobacterium*, *Arthrobacter* sp., *Proteus* sp., *Bacillus* sp., *Fusarium* sp., *Pseudomonas* sp., *Alternaria* sp., and *Laccaria* sp., and promote plant growth [103,104]. The compounds released by these organisms are specific to different metabolic pathways and play a key role in signaling a range of plant physiological processes and promoting growth related to the modulation of essential nutrients, hormonal balance, metabolism, and sugar concentrations [100]. Most of these studies were carried out with *A. thaliana* and reinforced the efficiency of using VOCs to promote plant growth [101,102,103,104].

Some researchers have already demonstrated the efficiency of these compounds in promoting growth and mitigating stress in plants [105,106,107], but most of these studies are carried out under controlled laboratory conditions. However, VOCs are still little used in agriculture, as these compounds have high biodegradability and reactivity. In addition, further research is needed on these compounds, the mechanism of perception in plant tissues, application techniques, and detailed identification of these molecules [101].

### 4.5. Bacterial Protection and Repairing Mechanism in Plant Tissue against Drought Stress

Reactive oxygen species (ROS) increase within subcellular compartments under drought stress, generating free radicals and redox imbalances (oxidative damage) that damage structural and functional macromolecules (e.g., biological membranes), compromising plant homeostasis. 

Antioxidant enzymes have a pivotal role in the response to water scarcity in plant tissues, leading to differential drought stress tolerance [37,40,41]. Bacterial inoculants have been recognized as modulators of plant antioxidant enzymes, enhancing crop protection by decreasing ROS levels. Several reports have shown increased activity of enzymatic antioxidants (ascorbate peroxidase, catalases, peroxidases, glutathione reductase, superoxide dismutase) as well non-enzymatic oxidants (e.g., ascorbic acid, flavonoids, and phenolic compounds) under stressful condition [108,109,110,111].

ROS accumulation impairs photosynthetic activity, compromising the antenna harvesting complex integrity, electron transport and enzyme functionality, and the chloroplast membrane system. The photosynthetic performance of *Glycyrrhiza uralensis* was improved by a plant growth-promoting *Bacillus pumilus*. Microscopy analysis revealed that the bacterium inoculation maintained the integrity of chloroplast and mitochondria cell structure under drought stress [112]. 

A boosted antioxidant plant response can be achieved by combining beneficial bacteria with bioactive products. For example, drought stress recovery in sugarcane was improved by combining endophytic diazotrophic bacteria with humic acids [89]. The bacterial inoculant induced the preservation of the water leaf potential and higher relative water content, and humic acids mitigated water stress by inducing antioxidant enzyme activity.

## 5. Microbial Inoculants to Mitigate Drought Stress in Agroecosystems

The massive use of industrial fertilizer obtained mainly from non-renewable resources is currently a severe problem for the environment as it is an essential contributor to the degradation of the ozone layer, emission of greenhouses gases into the atmosphere, and low-efficiency recovery by plants, as well as the high cost of its production. As a result, bioinoculants and other biological products pavemented their strategic importance as sustainable technologies for reducing chemical industrial fertilizers and pesticides, reducing the economic, social, and environmental impact of agriculture activity on the local-global levels [113].

Microbial inoculants designed as agricultural bioinputs are formulations composed of live microorganisms (fungi, bacteria, and algae) and/or their metabolites with biofertilizer, biostimulation, and bioprotection properties applied to agroecosystems as sustainable approaches [12,73,113]. Bioinoculants can be applied to soil, seeds, or plant surfaces (delivering niches) in distinct physicochemical formulations (microbial composition, carriers, and additives) and with proper time application that considers the physiological status of the crop (ontogeny-time delivery). 

In turn, these microorganisms can colonize the rhizosphere, surfaces, or interior of plants and promote plant growth by (a) increasing the nutrient availability in the plant-soil system (i.e., biological nitrogen fixation, mineral solubilization, and organic compounds mineralization) and (b) enhancing nutrient absorption by hormonal action (auxin, cytokinin, gibberellin, abscisic acid) that drives morpho-physiological changes in the plant host for increased nutrient use efficiency [12,53,73]. 

The commercial bioproducts used as microbial inoculants for agriculture were launched by Nobbe and Hiltner (1895) who introduced “Nitragin,” based on a rhizobia strain. Later, other products containing diazotrophic prokaryotes were developed based on *Azotobacter* and algae. However, it is known that numerous legume nodule-forming symbiotic bacteria, such as effective strains of the genera *Rhizobium*, *Bradyrhizobium*, *Sinorhizobium*, *Mesorhizobium*, as well a group generically identified as beta-rhizobia and non-nodulating bacterial genera such as *Azoarcus*, *Gluconacetobacter diazotrophicus*, *Azotobacter*, *Azospirillum*, *Paraburkholderia*, *Enterobacter*, *Pseudomonas,* and *Herbaspirillum*, among others, stand out for their ability to fix atmospheric nitrogen and/or secrete bioactive compounds are commonly used as bioinoculants [56]. In addition, several worldwide studies have reported the use of microorganisms formulated as bioinoculants for plant growth promotion in a wide range of crops under field conditions, such as sugarcane, rice, soybean, bean, chickpeas, tomatoes, maize, tropical fruits, and wheat, among others [12,79,114,115,116,117,118]. 

The adoption of bioinoculants by farmers is rapidly increasing [12], leveraging innovation and technologies to fulfill the bioproduct market. The Brazilian market of microbial products used as biofertilizers mainly consists of *Bradyrhizobium* spp. and *Azospirillum brasilense* applied to soybean and maize crops [12], respectively. Biological nitrogen fixation and the increased uptake of nutrients by roots are the primary plant growth-promotion modes of action. However, commercial inoculants offer side effects such as an “increase in the absorption of water and saline stress” or “produces phytohormones that promote more significant development of the root system, which results in increased absorption of water and nutrients, confers improved resistance to stresses such as salinity and drought.” 

Complementary to microbial inoculation actions on improving soil nutrient availability and root uptake, some mechanisms do not rely directly on promoting plant growth promotion effects. Nevertheless, they play an essential role in the response to adverse environmental conditions, mitigating the effects of abiotic and biotic stresses and promoting plant protection. These microbial mechanisms include ACC deaminase activity, ROS-enzymes synthesis, EPSs, volatile organic compounds and osmolyte production, and induced systemic resistance (ISR), among others that are less explored [36,45,61,80,94,99,119].

Thus, there are plenty of opportunities to design microbial inoculant targets to increase plant tolerance to drought, driven by plant–bacteria interaction attributes that cover a combination of water use efficiency (phytohormonal balance) and stress protection repair mechanisms. For example, a recently launched commercial product Auras^®^ (Embrapa and NOOA Ciência e Tecnologia Agrícola, Minas Gerais, Brazil)) formulated with *Bacillus aryabhattai* strain CMAA 1363 [120] represents an elegant and new technological solution to maximize the microbial potential and alleviate the drought effect on agroecosystems (https://www.embrapa.br/busca-de-solucoes-tecnologicas/-/produto-servico/4446/mitigacao-da-seca-por-bacterias-beneficas, URL accessed on 13 February 2023).

In earlier technological attempts, the candidate bacteria strains were screened from a bacteria collection obtained in a non-selective pressure environment. Then, under laboratory conditions, using assays involving water activity reduction (i.e., use of polyethylene glycol, salts, and other osmotic active molecules) [96,108] or studies on progressive cell-bacterium dissection [121], better-performing isolates were screened and further evaluated in the greenhouse and an open field. In parallel, plant-growth-promoting and those traits involved with water deficit tolerance have been considered addictive traits in bacterial selection programs.

A new generation of the most-effective microbial products has emerged based on microbe-driven-prospection for the rhizosphere, rhizoplane, and inner tissues of plant species adapted to harsh environments. An increased number of studies on the phenological traits of bacteria cells tolerant to abiotic stresses have been emerging [122]. By driven selection, a halotolerant PGPR could produce auxin and ACC-deaminase and induce salinity stress tolerance by secondary metabolites in tomatoes [123]. The whole genome analysis of bacterial candidates for inoculant formulations can assist in phenotypic screening. This was the case for *Bacillus altitudinis* (strain FD48), which was previously demonstrated to be an effective inducer of antioxidant stress in rice under drought and also presents a set of genes related to distinct mechanisms of water stress evaluation [124]. 

New approaches have been designed for the most-effective microbial products for drought stress. For example, Jochum and coworkers [125] proposed a bacterial bioprospecting screen coupling efficient root colonization and drought stress mediation driven to the cereal plant host. The onset procedure involved: (a) PGPB selection on rhizospheres of perennial grasses in a semi-arid environment, (b) a laboratory pre-screening focused on desirable plant phenotypes (delayed symptoms of water scarcity), and (c) a final selection of elite bacterial isolates (rapid colonizers and adequate stress crop protection) that can be formulated and delivered as soon as water stress is detected on the field [126]. 

Another successful bioprospecting-driven approach resulted in a Brazilian commercial bacterial inoculant recommended to alleviate plant water stress. Under an open-innovation business ecosystem, a group of researchers led by Melo [120] prospected cacti-associated bacteria from semi-arid environments and screened rhizobacteria for plant growth promotion under drought. Later, one strain of *Bacillus aryabhattai* (CMAA) was selected and, in collaboration with NOOA Ciência and Tecnologia Agrícola enterprise, one liquid formulation was developed and made available for farmers. The product brochure points out that the bacterial inoculant optimizes crop water use efficiency and a faster resumption of the production cycle after water stress events. 

## 6. Final Considerations

A series of responses are triggered for plant survival and resilience under stress conditions, and drought tolerance results from interplayed physiological, biochemical, and molecular complex network responses. The well-described core mechanisms can be divided into plant water conservation mechanisms and protection and damage restoration mechanisms. In addition, plant growth-promoting bacteria have been widely described as a tool to mitigate drought stress in plants. However, even recognizing the scientific advancement, it is necessary to have a more integrative and deeper understanding of these response mechanisms triggered by microorganisms and thus make it possible to increase crop yields using techniques and strategies that support water deficit.

On the other hand, the present technological knowledge allows us to offer a solution to design, formulate, and apply bacterial inoculants to increase plant resilience to drought stress. To our best knowledge, microbial bacteria represent a feasible solution to mitigate the adverse effects and the decrease in agricultural productivity.

Bacterial inoculants designed for drought stress mitigation should consider the main mechanisms underlined to increase the plant–microbe interaction under drought tolerance. We know that field conditions affect bacterial survival and inoculum efficiency. Thus, bacteria have several defense mechanisms to maintain their survival, such as the accumulation of osmoprotectants, antioxidant responses, expression of stress-related genes, and essential proteins to maintain cell viability. Advances in the molecular characterization of the responses triggered by drought and the identification of hormonal homeostasis are required, since microorganisms can produce and/or modulate several hormonal classes associated with changes in the concentration, location, and signaling of hormones and, consequently, affect the concentration and its balance in the plant.

Another challenge involves ABA signaling and the plant structural changes that increase plant water content in inoculated plants under water scarcity. Knowing that abscisic acid is one of the first signs of plant response under stress conditions, it is worth questioning whether the inoculation of BPCV affects the hormonal balance of ABA in plants under water stress conditions to increase the response of plants under drought conditions or increases agricultural productivity. This information can be used to select stress-tolerant microorganisms and enhance the use of BPCVs to mitigate the damage observed in agricultural production systems susceptible to water stress.

A new generation of bacterial inoculants driven to mitigate water stress in plants would benefit from the recent initiatives involving bacterial bioprospection under proper selective pressure (arid environments)—for example, the distinct soil–plant compartments (rhizosphere, rhizoplane, and inner tissue) under intense selective pressure constant water deficit [120,126]. The selection of bacteria strains with a superior ability to produce exopolysaccharides (EPSs) under osmotic stress in combination with batch reactor growth media and inoculant formulations that stimulated EPS secretion [125]. The microenvironment-rich EPSs that create a favorable niche for bacteria survival and root protection by trapping water and reducing system desiccation [127]. The design of inoculant formulations containing synthetic microbial communities based on compositional and functional metataxonomic and metagenomic data from plant microbiomes built under a drought stress environment [52] and the use of proper formulations that contain additives or carries that increase bacterial survival or display protective effects on plant tissues against abiotic stressors (i.e., humic substances) [128].

## Figures and Tables

**Table 1:** General mechanisms of drought resistance and bacterial genera/species.

Bacteria	Crop	Action Mechanism	Ref.
*Bacillus altitudinis*	Rice	Increment of secondary metabolites	[58]
*Bradyrhizobium diazoefficiens*	Soybean	GSR controlled biosynthesis of trehalose	[59]
*Azospirillum* sp.	Wheat	Highest amounts of N and auxin, with P solubilizing, ACC-deaminase activities	[60]
*Bacillus* sp.	Grass	Responses of antioxidant system and early proline accumulation	[61]
*Streptomyces* sp.	Tomato	Increase the content of different sugars and the RWC in leaves	[62]
*Burkholderia phytofirmans*	Maize	Improve ionic balance, antioxidant levels, and uptake of nitrogen	[63]
*Pseudomonas* sp.	Arabidopsis	Higher ACC deaminase activity, gibberellic acid, abscisic acid, indole acetic acid, and exopolysaccharide	[64]
*Enterobacter* sp. and *Leclercia adecarboxylata*	Bean	Enhance proline, malondialdehyde, and antioxidant enzymes	[65]
*Azospirillum brasilense* and *Stenotrophomonas maltophilia*	Wheat	Less accumulation of H_2_O_2_ with less enhanced production of proline and activities of catalase and peroxidase	[66]
*Herbaspirillum* sp. and *Azospirillum* sp.	Wheat	Higher relative plant tissue water content and better osmoregulation	[67]
